# Socioeconomic status and biomedical risk factors in migrants and native tuberculosis patients in Italy

**DOI:** 10.1371/journal.pone.0189425

**Published:** 2017-12-18

**Authors:** Silvia Pittalis, Pierluca Piselli, Silvia Contini, Gina Gualano, Mario Giuseppe Alma, Marina Tadolini, Pavilio Piccioni, Marialuisa Bocchino, Alberto Matteelli, Stefano Bonora, Antonio Di Biagio, Fabio Franzetti, Sergio Carbonara, Andrea Gori, Giovanni Sotgiu, Fabrizio Palmieri, Giuseppe Ippolito, Enrico Girardi

**Affiliations:** 1 Istituto Nazionale per le Malattie Infettive Lazzaro Spallanzani, Roma, Italy; 2 Ospedale San Camillo Forlanini, UOC Tisiologia e Broncopneumologia, Roma, Italy; 3 Alma Mater Studiorum Università di Bologna, Dipartimento di Scienze Mediche e Chirurgiche, UO Malattie Infettive, Bologna, Italy; 4 ASL Città di Torino, S.C. Pneumologia, Torino, Italy; 5 Università degli Studi Federico II, Dipartimento di Medicina Clinica e Chirurgia, Sezione di Malattie dell'Apparato Respiratorio, Napoli, Italy; 6 Spedali Civili di Brescia, Dipartimento di Scienze Cliniche e Sperimentali, Clinica Malattie Infettive, Brescia, Italy; 7 Università degli Studi di Torino, Dipartimento di Scienze Mediche, Clinica di Malattie Infettive, Torino, Italy; 8 Ospedale Policlinico San Martino, Clinica di Malattie Infettive, Genova, Italy; 9 Ospedale Sacco-Università di Milano, Malattie Infettive, Milano, Italy; 10 Policlinico Universitario Bari, Malattie Infettive, Bari, Italy; 11 Ospedale San Gerardo–Università di Milano Bicocca (Monza), Clinica di Malattie Infettive, Monza, Italy; 12 Università degli Studi di Sassari, Dipartimento di Medicina Clinica e Sperimentale, Sassari, Italy; National Institute of Health, ITALY

## Abstract

Action on social determinants is a main component of the World Health Organization End Tuberculosis (TB) Strategy. The aim of the study was to collect information on socioeconomic characteristics and biomedical risk factors in migrant TB patients in Italy and compare it with data collected among Italian TB patients. A cross-sectional study was conducted among TB patients aged ≥18 years over a 12-months enrolment period in 12 major Italian hospitals. Information on education, employment, housing and income was collected, and European Union Statistics on Income and Living Conditions index was used to assess material deprivation. Among migrants, we also analyzed factors associated with severe material deprivation. Migrants were compared with younger (18–64 years) and older (65+ years) Italians patients. Out of 755 patients enrolled (with a median age of 42 years, interquartile range: 31–53), 65% were migrants. Pulmonary, microbiologically confirmed, and new cases were 80%, 73%, and 87% respectively. Prevalence of co-morbidities (i.e. diabetes, chronic kidney disease, neoplastic diseases and use of immunosuppressive drugs) was lower among migrants compared to Italian TB patients, while indicators of socioeconomic status, income and housing conditions were worst in migrants. Forty-six percent of migrants were severely deprived vs. 9% of Italians (p<0.0001, 11.3% and 5.5% among younger and older Italians, respectively). Among migrants, being male, older, irregular, unemployed, with a shorter time spent in Italy, a lower education level, and without a co-morbidity diagnosis were factors associated with severe material deprivation at multi-variable logistic regression. Moreover, socioeconomic indicators for Italian patients did not differ from those reported for the general Italian population, while migrant TB patients seem to have a higher prevalence of severe material deprivation than other migrants residing in Italy. Intervention to address the needs of this population are urgent.

## Introduction

Tuberculosis (TB) in migrant populations represents a global priority given its profound health and social consequences [1]. Migrants, especially those moving from high TB incidence countries, are at increased risk of TB disease for different reasons including the risk of infection in home countries, health vulnerability related to pre-migration events, poor travel conditions, and poor living conditions in host communities [2]. Although the risk posed by migrants with TB to autochthonous populations seems low, they contribute disproportionately to the national TB burden in low TB incidence countries [3]. In these countries, TB incidence is generally declining, although at the current pace the achievement of the pre-elimination threshold (defined as less than 1 case per 100.000 inhabitants) will not be possible without specific interventions that address the needs of migrant populations [4–6].

The strict relationship between poverty and the risk of TB has been historically recognized [7] and recent studies show that socioeconomic factors remain critical drivers in the epidemiology of TB. Ecological studies show that income levels per capita, income inequality and spending on social protection are associated with TB burden [8–9]. Socioeconomic development appears to be an important determinant of the declining trends of TB observed in many countries in the past decade [10] and the burden of TB has been found to be associated with socioeconomic status both in low incidence [11–12] and high incidence countries [13–14]. Social factors may increase susceptibility to TB infection and disease, and may worsen its clinical outcome [15].

The fight against the social determinants of the disease constitute a pillar of the new World Health Organization (WHO) End TB Strategy [16] and interventions in this field need to be adapted and focused on the needs of migrants [1]. Migrants are heterogeneous populations whose living conditions vary considerably in the host country [17–18], and knowledge of social and behavioural factors potentially associated to the TB risk is needed to adapt policies and supportive systems to the unmet needs of migrants.

In this paper, we present the results of a multi-centre survey conducted in Italy, a low TB incidence country with an increasing proportion of cases in foreign born persons. The study was aimed at collecting information on socioeconomic characteristics and biomedical risk factors on migrant TB patients and comparing these data with those recorded in Italian TB patients. The information collected provide also the basis for comparison of selected socioeconomic indicators of TB patients with those collected for general population and for foreign born individuals in Italy.

## Material and methods

We conducted a survey on adult TB patients (18 years or older) diagnosed in 12 public hospitals in 10 Italian cities located in Northern, Central, and Southern Italy. Each participating centre enrolled patients consecutively observed in a 12-month period within a three-years period (2013–2015). The WHO case definitions were used to define and classify TB cases [19].

The survey design was based on the conceptual framework on social determinants of health developed by the WHO Commission on Social Determinants of Health, and on the framework on TB risk factors and determinants proposed by Lonnroth K et al. [20–21]. Briefly, these frameworks identify structural and upstream determinants of health at individual and household level, including socioeconomic status, education, occupation, and income, as well as intermediate determinants or proximate risk factors (e.g., material circumstances, behavioural, biological, and psychosocial factors). In particular, TB proximate risk factors are living conditions increasing the risk of exposure to *Mycobacterium tuberculosis* and biological and behavioural factors increasing the risk of developing TB disease.

For each patient, we collected information on the following data through an interview (See S1 Table for the list of items included): education, occupation, housing conditions, access to healthcare and material/living conditions. Indicators were chosen to allow comparisons with data collected by census or specific surveys carried out by the Italian National Institute of Statistics [22]. To assess material/living conditions, the European Statistics on Income and Living Conditions (EU-SILC) deprivation index was used. The EU-SILC project is the reference source for statistics and indicators on income and living conditions established under ‘framework’ Regulation No 1177/2003 of the European Commission to analyse and compare aspects of poverty in and across European Union (EU) Member States [23]. It is a multi-purpose instrument that focuses mainly on income, collecting detailed income components at household and individual level, but also gathers information on social exclusion, material deprivation, housing conditions, labour market participation, education and health [24]. In our analysis, the household monthly income was self-reported in the interview ranked using a 500€ step-increase up to 3000€ or above. We used in the analysis categorizing patients as poor if their household income was lower than 500€, which, regardless the number of persons in the household, is below the threshold of absolute poverty in current National statistics [22]. A person was defined as severely materially deprived according to EU-SILC if he/she experiences at least 4 out of 9 deprivation items in three domains: economic strain, durables and housing [25]. Regarding the education, we recorded the highest International Standard Classification of Education (ISCED) level attained [26].

We also collected from clinical files data on medical conditions and behaviours associated to an increased TB risk from clinical files: smoking tobacco, use of illicit drugs, Human Immunodeficiency Virus (HIV) infection, diabetes, chronic kidney disease, malnutrition, silicosis, malignancy, or use of immunosuppressive drugs [21,27].

*Migrants* were defined those patients who were not born in Italy, and irregular migrants were those whose movement took place outside the regulatory norms of the sending and transit countries and outside Italian regulations [28]. Italians patients were classified as younger (18–64 years, labour force) or older adult patients (65 years or above, mostly retired) according to the standard categorization used in national reports on indicators of socio-economic status [22].

Data were anonymized and imputed using an *ad hoc* on-line database hosted in a central server for further data management.

### Statistical analysis

Descriptive analysis was conducted to characterize subjects enrolled in the study. Median values and interquartile ranges (IQR) were used to describe numerical variables, while counts and percentages were employed for qualitative variables. The association between categorical variables and the different population groups (migrants versus the two distinct age-group of Italians) was assessed using chi-squared test or Fisher’s exact test, as appropriate. Mann-Whitney test was used to assess differences in the distribution of numeric variables between groups. The univariate association between severe deprivation and selected characteristics among migrants was assessed by means of odds ratios (OR) and their 95% confidence intervals (CI). Finally, we constructed a multi-variable final model adjusting for all variables found to be significantly associated with severe deprivation at univariate analysis (p<0.10) forcing age. A p-value less than 0.05 was considered statistically significant. Data management and analysis analyses were performed using IBM SPSS Statistics version 23 (IBM Corp., Armonk, NY, USA) or STATA version 13 (StataCorp 2013, College Station, TX, USA: StataCorp LP).

The study was approved by the Ethical Committees of the coordinating (i.e., National Institute for Infectious Diseases “Lazzaro Spallanzani”, Rome) and participating centers. All enrolled patients provided written informed consent.

## Results

Out of 812 newly detected TB cases diagnosed in the participating centers during the study period, 755 (93.0%) patients were enrolled.

The main characteristics of the study population are summarized in Table 1. The patients were mostly males (61.3%) with a median age of 42 years (IQR: 31–53). New TB cases were 87.2%, pulmonary TB cases were 80.4%, and pulmonary bacteriologically confirmed were 75.1% (72.8% among all 755 TB cases). More than two-third of the recruited patients were migrants (522, 69.1%), whit a median (IQR) time since arrival in Italy of 7.6 (3.6–12.5) years. Migrants were more likely to be male (64.2% vs. 54.9%) and younger than Italians TB patients (median age 37 vs. 57 years). The area of origin most represented was Eastern Europe (45.6% of all migrants), and Romania, Morocco and Philippines were the most represented countries of origin (38.9%, 8.2% and 5.0% respectively). More than 10% were irregular migrants.

**Table 1:** Characteristics of 755 patients with tuberculosis according to the geographical origin.

		Total (N = 755)	Migrants (N = 522)	Italians (N = 233)
Characteristics		N (%)	N (%)	N (%)
Sex	Male	463 (61.3)	335 (64.2)	128 (54.9)
Age groups (years)	18–44	426 (56.4)	365 (69.9)	61 (26.2)
	45–64	224 (29.7)	143 (27.4)	81 (34.8)
	≥65	105 (13.9)	14 (2.7)	91 (39.1)
Site of TB disease	Pulmonary	607 (80.4)	422 (80.8)	185 (79.4)
TB Case definition	Clinically diagnosed	167 (22.1)	121 (23.2)	46 (19.7)
	Bacteriologically confirmed	550 (72.8)	376 (72.0)	174 (74.7)
	Unknown or missing	38 (5.0)	25 (4.8)	13 (5.6)
TB classification according to previous TB treatment	New case	658 (87.2)	453 (86.8)	205 (88.0)
Area of origin	Italy	233 (30.9)	-	233 (100)
	Eastern Europe	238 (31.5)	238 (45.6)	-
	Latin-America	44 (5.8)	44 (8.4)	-
	South-East Asia	97 (12.8)	97 (18.6)	-
	Africa	139 (18.4)	139 (26.6)	-
	Other	4 (0.5)	4 (0.8)	-
Years in Italy	Median (IQR)		7.6 (3.6–12.5)	
Legal status	Irregular		61 (11.7)	
	Regular, non-EU citizen		227 (43.5)	
	Regular, EU citizen		234 (44.8)	

N, number; TB, tuberculosis; IQR, Interquartile Range; EU, European Union.

Indicators of socioeconomic status are shown in Table 2. Educational level was similar in migrants and in younger Italian patients, and higher in migrants than in elderly Italian patients. All other indicators were, in general, significantly worse in migrant patients. With respect to the employment status, proportion of individual unemployed or occasionally employed was higher among migrants compared to younger Italian patients. Regarding the housing conditions, migrants were more likely to be homeless or living in a shelter/tent/motor home (6.3% vs. <1%), and to live in crowded households than Italian TB patients

**Table 2:** Selected indicators of socioeconomic status in 755 patients with tuberculosis according to the geographical origin.

Characteristics			Migrants (N = 522)	Italians <65 years (N = 142)		Italians 65+ years (N = 91)	
			N (%)	N (%)	*p* value*	N (%)	*p* value*
Highest ISCED level attained	Secondary or above	328 (62.9)	83 (58.5)	NS	33 (36.3)	<0.001
	Lower secondary	111 (21.3)	46 (32.4)		21 (23.1)	
	Primary education	79 (15.1)	11 (7.7)		35 (38.5)	
	Unknown or missing	4 (0.8)	2 (1.4)		2 (2.2)	
Position in the labour marker	Unemployed	221 (42.3)	43 (30.3)	<0.001	-	<0.001
	Retired or Inactive	3 (0.6)	6 (4.2)		79 (86.6)	
	Employed	156 (29.9)	61 (43.0)		4 (4.4)	
	Occasionally employed	117 (22.4)	13 (9.2)		-	
	Self-employed	20 (3.8)	17 (12.0)		4 (4.4)	
	Other or Unknown	5 (1.0)	2 (1.4)		4 (4.4)	
Housing condition	House/Apartment	453 (86.8)	140 (98.6)	<0.001	87 (95.6)	<0.01
	Collective housing	34 (6.5)	1 (0.7)		4 (4.4)	
	Homeless/living in a shelter	33 (6.3)	1 (0.7)		-	
	Unknown or missing	2 (0.4)	-		-	
Persons in the household^a^	<5	347 (76.6)	125 (89.3)	<0.001	86 (98.9)	<0.001
	≥5	95 (21.0)	15 (10.7)		1 (1.1)	
	Unknown or missing	11 (2.4)	-		-	
N. of persons in households per room^a^	Median (IQR)	1.3 (1.0–2.0)	0.8 (0.5–1.0)	<0.001	0.7 (0.4–1.0)	<0.001
	Unknown or missing	17	1		2	
Unavailability of (1 or more):	Yes	80 (15.3)	20 (14.1)	NS	2 (2.2)	<0.001
running drinkable water/heating/bathroom^a^	Unknown or missing	2 (0.4)	-		-	
History of imprisonment	Yes	26 (5.0)	12 (8.5)	NS	1 (1.1)	NS
	Unknown or missing	14 (2.7)	6 (4.2)		8 (8.8)	
EU-SILC deprivation Index	≥4 (highly deprived)	238 (45.6)	16 (11.3)	<0.001	5 (5.5)	<0.001
	Unknown or missing	10 (1.9)	-		-	

N, number; ISCED, International Standard Classification of Education; IQR, Interquartile Range; NS: not significant (p>0.05).

^a^ These data were calculated only on subjects living in house or apartment (N = 680).

* The data in these columns indicate the significativity of the comparison with migrants’ characteristics. Differences between groups were examined with the chi-squared test for proportions and with Mann-Whitney U-test for continuous variables.

The household monthly income (data available for 650 patients, 86.1%) was significantly lower among migrant than in Italian TB patients (when compared with both age categories). The proportion of patients with a household monthly income lower or equal to 500€ was 23.2% among migrant patients vs. 7.7% and 2.2% among younger and older Italian patients, respectively (p<0.001).

Overall, the proportion of migrants with a condition of severe material deprivation (45.6%) was four times higher than in younger Italian patients (11.3%) and more than eight times higher compared with elderly Italian patients (5.5%).

Table 3 reports data on biological and behavioural risk factors for TB. Smoking and intravenous drug use were less frequent among migrants, while the prevalence of HIV infection was similar in migrant and Italian younger patients. The frequency of use of immunosuppressive drugs and of diagnosis of malignancies was lower in migrants than in Italian patients, whereas the prevalence of diabetes mellitus or chronic kidney disease was similar in migrant and younger Italian patients, and lower in migrant than in elderly Italian patients. Overall, the prevalence of co-morbidities was lower among migrants compared to Italian patients.

**Table 3:** Selected behavioral and biological risk factors in 755 patients with tuberculosis according to the geographical origin.

Characteristics		Migrants (N = 522)	Italians <65 years (N = 142)		Italians 65+ years (N = 91)	
		N (%)	N (%)	*p* value*	N (%)	*p* value*
Smoking tobacco	No	262 (50.2)	47 (33.1)	<0.01	46 (50.5)	<0.001
	Yes, former	74 (14.2)	22 (15.5)		33 (36.3)	
	Yes, current	183 (35.1)	71 (50.0)		11 (12.1)	
	Unknown or missing	3 (0.6)	2 (1.4)		1 (1.1)	
Use of illicit drugs	No	486 (93.1)	120 (84.5)	<0.001	87 (95.6)	NS
	Yes, IVDU	2 (0.4)	4 (2.8)		-	
	Yes, Other	27 (5.2)	16 (11.3)		1 (1.1)	
	Unknown or missing	7 (1.3)	2 (1.4)		3 (3.3)	
HIV infection	Test performed, Positive	20 (3.8)	6 (4.2)	NS	1 (1.1)	<0.001
	Test performed, Negative	421 (80.7)	108 (76.1)		53 (58.2)	
	Not performed or unknown	81 (15.5)	28 (19.7)		37 (40.7)	
Other co-morbidities	Diabetes	33 (6.3)	14 (9.9)	NS	20 (22.0)	<0.001
	Chronic kidney disease	6 (1.1)	1 (0.7)	NS	11 (12.1)	<0.001
	Malnutrition	7 (1.3)	9 (6.3)	<0.01	2 (2.2)	NS
	Silicosis	1 (0.2)	1 (0.7)	NS	0 (0.0)	NS
	Malignancy	9 (1.7)	9 (6.3)	<0.01	15 (16.5)	<0.001
	Receiving immunosuppressive treatment	14 (2.7)	19 (13.4)	<0.001	20 (22.0)	<0.001
	Any of the other co-morbidities^a^	61 (11.7)	43 (30.3)	<0.001	44 (48.4)	<0.001

N, number; IVDU: intravenous drug user; HIV, human immunodeficiency virus; NS: not significant (p>0.05).

^a^ As diabetes, chronic kidney disease, malnutrition, silicosis, malignancy or receiving immunosuppressive treatment.

* The data in these columns indicate the significativity of the comparison with migrants’ charactheristics. Differences between groups were examined with the chi-squared test for proportions and with Mann-Whitney U-test for continuous variables

Finally, Table 4 reports the analysis of factors associated with severe material deprivation among 512 migrant patients for whom this information was available. At univariate analysis, severely deprived migrants were more likely to be males, older, irregular, unemployed, with a shorter time spent in Italy and a lower education level; conversely, they were less likely to have a co-morbidity or HIV infection. These associations were confirmed at the multi-variable analysis.

**Table 4:** Association of selected factors with severe material deprivation (according to the EU-SILC deprivation index) in 512 migrants with tuberculosis.

Characteristics		All	Severe deprived	OR (95% CI)	aOR (95% CI)
		N	N (%)		
		512	238 (46.5)		
Sex	Females	185	66 (35.7)	1	1
	Males	327	172 (52.6)	2.00 (1.38–2.90)*	2.02 (1.34–3.05)*
Age (years)	<45	356	159 (44.7)	1	1
	≥45	156	79 (50.6)	1.27 (0.87–1.85)	1.59 (1.01–2.49)*
Highest ISCED level attained	Secondary or above	327	125 (38.2)	1	1
	Lower secondary	105	57 (54.3)	1.92 (1.23–2.99)*	1.71 (1.06–2.74)*
	Primary education	76	54 (71.1)	3.97 (2.30–6.83)*	3.10 (1.72–5.60)*
	Unknown or missing	4	2 (50.0)	1.62 (0.23–11.62)	0.95 (0.13–7.20)
Number of years in Italy	<5	169	97 (57.4)	1	1
	5–10	142	65 (45.8)	0.63 (0.40–0.98)*	0.92 (0.56–1.51)
	>10	193	70 (36.3)	0.42 (0.28–0.65)*	0.56 (0.35–0.92)*
	Unknown	8	6 (75.0)	2.23 (0.44–11.36)	2.56 (0.43–15.10)
Legal status	Regular, non-EU citizen	221	94 (42.5)	1	1
	Regular, EU citizen	232	103 (44.4)	1.08 (0.74–1.56)	1.19 (0.79–1.80)
	Irregular	59	41 (69.5)	3.08 (1.66–5.69)*	2.05 (1.05–4.01)*
Co-morbidities ^a^	No	434	210 (48.4)	1	1
	Yes	78	28 (35.9)	0.60 (0.36–0.98)*	0.43 (0.24–0.77)*
Position in the labour market	Employed or Retired	293	108 (36.9)	1	1
	Unemployed	219	129 (58.9)	2.50 (1.75–3.58)*	2.43 (1.61–3.66)*

N, number; ISCED, International Standard Classification of Education; OR, odds-ratio; aOR: multivariate logistic regression OR estimate, adjusted for all variables in the table; CI, confidence intervals.

^a^ As diabetes, chronic kidney disease, malnutrition, silicosis, malignancy or receiving immunosuppressive treatment, HIV-infection.

* p<0.05.

## Discussion

In this paper, we provided a profile of socioeconomic status, and behavioural and biological risk factors of migrant TB patients in comparison with autochthonous TB patients in Italy, a low incidence country. Prevalence of clinical or behavioural known risk factors for TB was lower among migrants compared to Italian patients. On the other hand, socioeconomic status among migrants, measured through different indicators, was significantly worse than that recorded among Italians, regardless of age. This finding was not unexpected since living conditions of migrants in European countries, and elsewhere, are generally worse than those of the natives [25,28], and the situation of foreigners living in Italy follows this general pattern [18,29]. However, our study suggests that, while socio-economic status in Italian TB patients did not differ from that reported for the general population, migrant TB patients may live in worse conditions in comparison with other migrants residing in Italy.

Many measures are used to assess poverty risk and deprivation, based on the evidence that poverty is a multidimensional phenomenon that encompasses both the individual’s and the household’s standard of living, in particular for migrant populations [18]. While considering only income or the proportion of those with a household income lower than a standard threshold for poverty could not be the best way to assess the living conditions of migrants, measures of subjective poverty and material deprivation better capture their socioeconomic status [18]. We used EU–SILC deprivation index in our study because it represents a standardized tool used in and across EU Member States for policy monitoring, allowing comparison over time and between the different countries. It is also widely used in Italy in national statistics reports, allowing us to compare our data with a measurable and reliable indicator, frequently updated at country-level [23].

Prevalence of severe deprivation for Italian TB patients (9%) was slightly lower than that reported at national level (11.5% in 2015) also when the patients were stratified by age (11.5 and 5.5 vs 12.2 and 8.3 at national level for younger and elderly patients respectively) [29]. In contrast, the prevalence of severe deprivation in migrant TB patients was 45.6% compared to 22.9% reported in Italy for household with foreigners [29]. Data on prevalence of severe deprivation according to different nationality group confirm this finding. We found that the prevalence of severe deprivation among TB patients from Romania, Morocco and Philippines (i.e. the groups most represented in our study) was 44.3%, 44.2% and 34.6% respectively, while those proportions in the same groups at national level were 12.8%, 31.9% and 15.5%, respectively, as shown by Busetta et al. in their elaboration based the Italian National Institute of Statistics 2009 survey on socioeconomic status among foreigners in Italy [18].

The effect of educational attainment on poverty and health are well investigated and some studies showed that higher levels of education are strongly associated to lower levels of persistent poverty [30–31]. In our study, the educational level of migrants and Italian younger patients was similar, and this observation is consistent with a previous study that showed a relatively comparable educational level in migrants and natives in Italy [32].

Indicators on vulnerability in the labour market showing discrepancies between Italian and migrant TB patients reflect national data, which show that migrants have higher rates of unemployment or occasional/short-term occupation than Italians [18].

Finally, overcrowding is a major factor in the transmission of respiratory infections by increasing the opportunity for cross infection [21]. In our analysis, the median number of persons in households per room and household crowding (inhabitants per 100 m^2^) were greater for migrant (1.3 and 5 respectively) than for <65 years (0.8 and 3.3) and >65 years (0.7 and 2) Italian TB patients, the latter similar to the national data (0.7 and 2.7 respectively) [29]. This may reflect the overall housing condition of migrants in Italy, as they usually live in rented or sub-let accommodations, which are frequently overcrowded [18,29,33],

Taken together, our results suggest that patients with TB may represent a particularly deprived subpopulation among migrants in our country. This is consistent with a previous study in an Italian metropolitan area which documented higher rates of TB among socially marginalized migrants [34] Moreover, our analysis shows that male migrant patients, those recently arrived in Italy, those less educated and those with an irregular migration status are at increased risk of experiencing severe material deprivation.

Older Italian patients had the highest prevalence of co-morbidity conditions in our study, consistently with the increase in age related prevalence of chronic illnesses that may impair defences against TB [35]. Furthermore, migrants had a lower prevalence of co-morbidities also when compared to younger Italian patients. The lower prevalence of these conditions among migrants can reflect the so called the ‘healthy migrant effect’, which implies that migration is selective for the healthiest individuals [36]. However, it is possible that some of these conditions are undiagnosed among migrants: the odds of experiencing unmet need for medical care was estimated 1.4 times higher for regular migrants and 2.5 times higher for irregular immigrants as compared with Italians, and the gap was even more striking with regard to chronic illnesses [18]. Based on our results, however, we can speculate that chronic co-morbidities may play a minor role in determining TB risk among migrants.

The main limitations of this study need to be noted. Our study was not conducted on a random sample of TB patients in Italy, and some regions were not represented. However enrolled patients represent roughly ¼ of the overall estimated number of 3500 TB cases diagnosed annually in Italy [37]. Demographic and clinical characteristics of enrolled patients, including the proportion of foreign born persons, are similar to those of TB patients notified in Italy during the same period [37]. Therefore, our data can be seen as broadly representative of the situation in our country and possibly of other European countries who share the same TB epidemiological situation. We did not collect information on the aspect of the pre-migration and migration process that may be relevant for the risk of TB. Finally, our study provides a socioeconomic profile of migrants TB patients, but it was not designed to analyze directly the role of socioeconomic status in determining the risk of developing TB in the migrant population. Previous ecological studies on this issue in low TB incidence countries provided conflicting results. A study on national United States TB surveillance data founded a weaker association between the TB rate and living in low socioeconomic status areas among migrants compared to natives [38]. No significant correlation was found between levels of deprivation and TB incidence in South Asian communities in the United Kingdom [39], while a study of TB notifications in the same country suggested that deprivation may be an important contributing factor for the high risk of developing TB at least in some migrant communities [40].

## Conclusions

Our study shows that a significant proportion of migrant patients with TB in Italy have a low socioeconomic status; this proportion is not only higher than that found among Italian patients, but, more importantly, higher than that found in the overall population of migrants living in Italy.

It remains to be determined to what extent a low socioeconomic status contributes to the risk of TB and on determining a poor TB outcome among migrants in Italy. There is evidence that financial support may improve TB outcome of some populations in low TB incidence countries [41] as well as for impoverished patients in countries with high TB incidence [42]. Studies assessing the effect of these interventions among migrants in low TB incidence countries are urgently needed along with interventions aimed at alleviating poverty among the most deprived migrants.

## Supporting information

S1 Table Summary of items included in the patient interview on social determinants of health. (PDF)
