# Supplementary material for: Socioeconomic status and biomedical risk factors in migrants and native tuberculosis patients in Italy
Source: PLoS One. 2017 Dec 18;12(12):e0189425. doi: 10.1371/journal.pone.0189425 (PMC5734730; doi:10.1371/journal.pone.0189425)
Supplement: S1 Table — (PDF) [file pone.0189425.s001.pdf]

**S1 Table. Summary of items included in the patient interview on social determinants of health.**

| |
|---|
| Education and highest-level attainment |
| • What is your highest level of education attained? |
| Migratory condition |
| • When you firstly arrived in Italy?<br>• Do you have a regular residency permit? |
| Employment status |
| • Do you have a job?<br>• What is your job?<br>• Do you have a permanent or non-permanent contract?<br>• Are you self-employed?<br>• Do you have a full-time or part-time contract?<br>• How many hours a week you work?<br>• Which is your monthly household income? |
| Housing Conditions/Accommodation |
| • Where do you live? Which is your accommodation (house, flat, shared household, bungalow, caravan or other mobile home, other)?<br>• Is there anyone else in your household? Who are your cohabitants?<br>• How many rooms do you have altogether in your accommodation, that's excluding bathrooms, toilets and kitchens?<br>• Have you got either a bath or a shower for sole use of the household?<br>• Do you have an inside flushing toilet for the sole use of the household?<br>• Is your accommodation kept adequately warm if needed?<br>• Do you have running drinkable water available?<br>• Do you have hot water? |
| Can you and your household have been able to: |
| 1. eat meat or proteins regularly (a meal with meat, chicken, fish - or vegetarian equivalent - every second day?<br>2. face unexpected financial expenses?<br>3. pay for one week’s annual holiday away from home?<br>4. to pay bills or any regular debt repayments?<br>5. to keep your home adequately warm? |
| Do you have for your private use/ for the sole use of the household: |
| 6. a car?<br>7. a washing machine?<br>8. a colour TV?<br>9. a telephone (including mobile phone)? |
